# Supplementary material for: A universal framework for accurate and efficient geometric deep learning of molecular systems
Source: Sci Rep. 2023 Nov 6;13:19171. doi: 10.1038/s41598-023-46382-8 (PMC10628308; doi:10.1038/s41598-023-46382-8)
Supplement: Supplementary file 1 — Supplementary Information. [file 41598_2023_46382_MOESM1_ESM.pdf]

Supplementary Information for "A Universal Framework for Accurate and Efficient Geometric Deep Learning of Molecular Systems"

Shuo Zhang[2,3]**, Yang Liu**[2]**, and Lei Xie**[1,2,3,*]

[1]Ph.D. Program in Computer Science, The Graduate Center, The City University of New York, New York, 10016, United States
[2]Department of Computer Science, Hunter College, The City University of New York, New York, 10065, United States
[3]Helen & Robert Appel Alzheimer's Disease Research Institute, Feil Family Brain & Mind Research Institute, Weill Cornell Medicine, Cornell University, New York, 10065, United States
[*]lei.xie@hunter.cuny.edu

Details of baselines

The following methods are being compared with our PAMNet in experiments:

Small molecule property prediction

- **SchNet**[1] is a GNN that uses continuous-filter convolutional layers to model atomistic systems. Interatomic distances are used when designing convolutions.
- **PhysNet**[2] uses message passing scheme for predicting properties of chemical systems. It models chemical interactions with learnable distance-based functions.
- **MGCN**[3] utilizes the multilevel structure in molecular system to learn the representations of quantum interactions level by level based on GNN. The final molecular property prediction is made with the overall interaction representation.
- **PaiNN**[4] is a GNN that augments the invariant SchNet into equivariant flavor by projecting the pairwise distances via radial basis functions and iteratively updates the geometric vectors along with the scalar features.
- **DimeNet++**[5] is an improved version of DimeNet[6] with better accuracy and faster speed. It can also be used for non-equilibrium molecular structures.
- **SphereNet**[7] is a GNN method that achieves local completeness by incorporating comprehensive 3D information like distance, angle, and torsion information for 3D graphs.

RNA 3D structure prediction

- **ARES**[8] is a state-of-the-art machine learning approach for identifying accurate RNA 3D structural models from candidate ones. It is a GNN that integrates rotational equivariance into the message passing.
- **Rosetta**[9] is a molecular modeling software package that provides tools for RNA 3D structure prediction.
- **RASP**[10] is a full-atom knowledge-based potential with geometrical descriptors for RNA structure prediction.
- **3dRNAscore**[11] is an all-heavy-atom knowledge-based potential that combines distance-dependent and dihedral-dependent energies for identifying native RNA structures and ranking predicted structures.

Protein-ligand binding affinity prediction

- **ML-based methods** include linear regression (LR), support vector regression (SVR), and random forest (RF). These approaches use the inter-molecular interaction features introduced in RF-Score[12] as input for prediction.
- **Pafnucy**[13] is a representative 3D CNN-based model that learns the spatial structure of protein-ligand complexes.

- **OnionNet**[14] is a CNN-based method that generates 2D interaction features by considering rotation-free element-pair contacts in complexes.
- **GraphDTA**[15] uses GNN models to learn the complex graph and utilizes CNN to learn the protein sequence. We use the best-performed variant (GAT-GCN) for comparison.
- **SGCN**[16] utilizes atomic coordinates and leverages node positions based on graph convolutional network[17].
- **GNN-DTI**[18] is a distance-aware graph attention network[19] that considers 3D structural information to learn the intermolecular interactions in protein-ligand complexes.
- **D-MPNN**[20] is a message passing neural network that incorporates edge features. The aggregation process addresses the pairwise distance information contained in edge features.
- **MAT**[21] utilizes inter-atomic distances and employs a molecule-augmented attention mechanism based on transformers for graph representation learning.
- **DimeNet**[6] is a message passing neural network using directional message passing scheme for small molecules. Both distances and angles are used when modeling the molecular interactions.
- **CMPNN**[22] is built based on D-MPNN and has a communicative message passing scheme between nodes and edges for better performance when learning molecular representations.
- **SIGN**[23] is a recent state-of-the-art GNN for predicting protein-ligand binding affinity. It builds complex interaction graphs for protein-ligand complexes and integrates both distance and angle information in modeling.

For small molecule property prediction, we use the baseline results reported in their original works for baselines. For RNA 3D structure prediction, we use the baseline results in[8]. For protein-ligand binding affinity prediction, we use the baseline results in[23]. When performing efficiency evaluation in our experiments, we adopt the public-available implementations of the related models: For DimeNet and DimeNet++, we adopt the implementation by PyTorch Geometric[24] at https://github.com/rusty1s/pytorch_geometric/blob/73cfaf7e09/examples/qm9_dimenet.py. For SphereNet, we use the official implementation at https://github.com/divelab/DIG. For ARES, we use the official implementation at https://zenodo.org/record/6893040. For SIGN, we use the official implementation at https://github.com/PaddlePaddle/PaddleHelix/tree/dev/apps/drug_target_interaction/sign.

Detailed analysis of near-native ranking task on RNA-Puzzles

For each RNA in RNA-Puzzles, we rank the structural models using PAMNet and four baseline scoring functions. For each scoring function, we select the $N \in \{1, 10, 100\}$ best-scoring structural models for each RNA. For each RNA, scoring function, and N, we show the lowest RMSD across structural models in Figure S1). The RMSD results are quantized to determine if each RMSD is below 2Å, between 2Å and 5Å, between 5Å and 10Å, or above 10Å. From the results, we find that for each RMSD threshold (2Å, 5Å, or 10Å) and for each N, the number of RNAs with at least one selected model that has RMSD below the threshold is greater when using PAMNet than when using any of the other four baseline scoring functions.

Statistical significance between SIGN and PAMNet on PDBbind

We use p-value to compute the statistical significance between SIGN and PAMNet on PDBbind. As shown in Table S1, PAMNet performs significantly better than SIGN on all four metrics with $p\text{-value} < 0.05$.

Detailed results of ablation study on QM9

In Table S2, we list the results of all properties on QM9 in our ablation study.

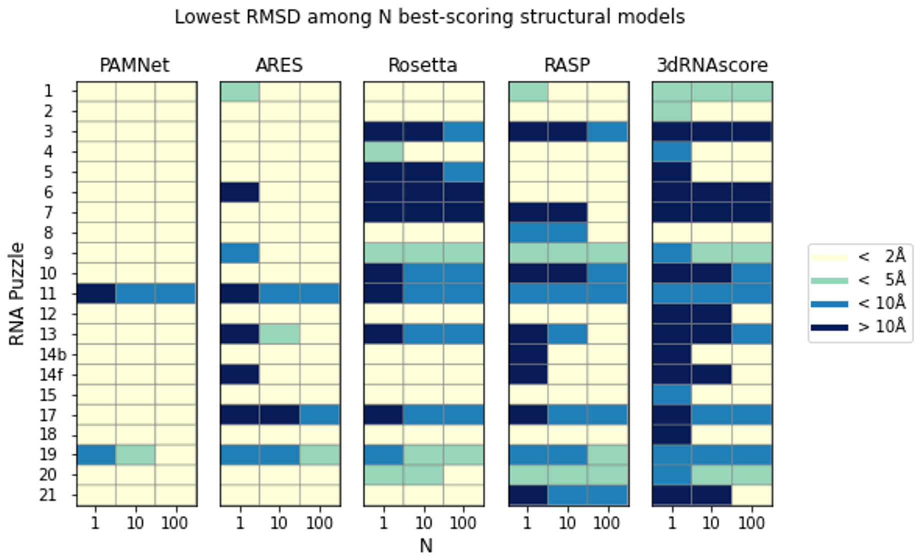

Figure S1. Detailed analysis of near-native ranking task on RNA-Puzzles. The results of the lowest RMSD among N best-scoring structural models of each RNA predicted by each scoring function are compared.

Model	RMSE ↓	MAE ↓	SD ↓	R ↑
SIGN	1.316 (0.031)	1.027 (0.025)	1.312 (0.035)	0.797 (0.012)
**PAMNet**	**1.263 (0.017)**	**0.987 (0.013)**	**1.261 (0.015)**	**0.815 (0.005)**
Significance (p-value)	0.0122	0.0156	0.0242	0.0212

Table S1. Statistical significance (p-value) between PAMNet and SIGN on PDBbind. The best results are marked in bold.

Model	μ	α	$\varepsilon_{\text{HOMO}}$	$\varepsilon_{\text{LUMO}}$	$\delta\varepsilon$	$\langle R^2 \rangle$	ZPVE	U_0	U	H	G	c_v
PAMNet	**10.8**	**0.0447**	**22.8**	**19.2**	**31.0**	**0.093**	**1.17**	**5.90**	**5.92**	**6.04**	**7.14**	**0.0231**
PAMNet w/o Attention Pooling	11.1	0.0469	24.2	20.3	32.8	0.094	1.22	6.12	6.15	6.29	7.44	0.0234
PAMNet w/o Local MP	13.9	0.0512	27.8	23.3	37.6	0.104	1.27	7.55	7.57	7.74	9.13	0.0262
PAMNet w/o Global MP	21.8	0.0887	41.5	34.9	56.4	5.53	1.52	8.80	8.81	9.01	10.6	0.0316

Table S2. Results of all properties on QM9 in ablation study.

Hyperparameters	Value		
	QM9	RNA-Puzzles	PDBbind
Batch Size	32, 128	8	32
Hidden Dim.	128	16	128
Initial Learning Rate	1e-4	1e-4	1e-3
Number of Layers	6	1	3
Max. Number of Epochs	900	50	100

Table S3. List of typical hyperparameters used in our experiments.

References

1. Schutt, K. *et al.* Schnetpack: A deep learning toolbox for atomistic systems. *J. chemical theory computation* **15**, 448–455 (2018).
2. Unke, O. T. & Meuwly, M. Physnet: A neural network for predicting energies, forces, dipole moments, and partial charges. *J. chemical theory computation* **15**, 3678–3693 (2019).
3. Lu, C. *et al.* Molecular property prediction: A multilevel quantum interactions modeling perspective. In *Proceedings of the AAAI Conference on Artificial Intelligence*, vol. 33, 1052–1060 (2019).
4. Schütt, K., Unke, O. & Gastegger, M. Equivariant message passing for the prediction of tensorial properties and molecular spectra. In *International Conference on Machine Learning*, 9377–9388 (PMLR, 2021).
5. Klicpera, J., Giri, S., Margraf, J. T. & Günnemann, S. Fast and uncertainty-aware directional message passing for non-equilibrium molecules. In *Machine Learning for Molecules Workshop at NeurIPS 2020* (2020).
6. Klicpera, J., Groß, J. & Günnemann, S. Directional message passing for molecular graphs. In *International Conference on Learning Representations* (2020).
7. Liu, Y. *et al.* Spherical message passing for 3d molecular graphs. In *International Conference on Learning Representations (ICLR)* (2022).
8. Townshend, R. J. *et al.* Geometric deep learning of rna structure. *Science* **373**, 1047–1051 (2021).
9. Watkins, A. M., Rangan, R. & Das, R. Farfar2: improved de novo rosetta prediction of complex global rna folds. *Structure* **28**, 963–976 (2020).
10. Capriotti, E., Norambuena, T., Marti-Renom, M. A. & Melo, F. All-atom knowledge-based potential for rna structure prediction and assessment. *Bioinformatics* **27**, 1086–1093 (2011).
11. Wang, J., Zhao, Y., Zhu, C. & Xiao, Y. 3drnascore: a distance and torsion angle dependent evaluation function of 3d rna structures. *Nucleic acids research* **43**, e63–e63 (2015).
12. Ballester, P. J. & Mitchell, J. B. A machine learning approach to predicting protein–ligand binding affinity with applications to molecular docking. *Bioinformatics* **26**, 1169–1175 (2010).
13. Stepniewska-Dziubinska, M. M., Zielenkiewicz, P. & Siedlecki, P. Development and evaluation of a deep learning model for protein–ligand binding affinity prediction. *Bioinformatics* **34**, 3666–3674 (2018).
14. Zheng, L., Fan, J. & Mu, Y. Onionnet: a multiple-layer intermolecular-contact-based convolutional neural network for protein–ligand binding affinity prediction. *ACS omega* **4**, 15956–15965 (2019).
15. Nguyen, T. *et al.* Graphdta: predicting drug–target binding affinity with graph neural networks. *Bioinformatics* **37**, 1140–1147 (2021).
16. Danel, T. *et al.* Spatial graph convolutional networks. In *Neural Information Processing: 27th International Conference, ICONIP 2020, Bangkok, Thailand, November 18–22, 2020, Proceedings, Part V*, 668–675 (Springer, 2020).
17. Kipf, T. N. & Welling, M. Semi-supervised classification with graph convolutional networks. *arXiv preprint arXiv:1609.02907* (2016).
18. Lim, J. *et al.* Predicting drug–target interaction using a novel graph neural network with 3d structure-embedded graph representation. *J. chemical information modeling* **59**, 3981–3988 (2019).
19. Veličković, P. *et al.* Graph attention networks. In *International Conference on Learning Representations* (2018).
20. Yang, K. *et al.* Analyzing learned molecular representations for property prediction. *J. chemical information modeling* **59**, 3370–3388 (2019).
21. Maziarka, Ł. *et al.* Molecule attention transformer. *arXiv preprint arXiv:2002.08264* (2020).
22. Song, Y. *et al.* Communicative representation learning on attributed molecular graphs. In *IJCAI*, 2831–2838 (2020).
23. Li, S. *et al.* Structure-aware interactive graph neural networks for the prediction of protein-ligand binding affinity. In *Proceedings of the 27th ACM SIGKDD Conference on Knowledge Discovery & Data Mining*, 975–985 (2021).
24. Fey, M. & Lenssen, J. E. Fast graph representation learning with PyTorch Geometric. In *ICLR Workshop on Representation Learning on Graphs and Manifolds* (2019).
